# The SHARP study: a quantitative and qualitative evaluation of the short-term outcomes of housing and neighbourhood renewal

**DOI:** 10.1186/1471-2458-9-415

**Published:** 2009-11-17

**Authors:** Mark Petticrew, Ade Kearns, Phil Mason, Caroline Hoy

**Affiliations:** 1 Public & Environmental Health Research Unit, London School of Hygiene & Tropical Medicine, London UK; 2 Department of Urban Studies, University of Glasgow, Glasgow, UK; 3 NHS Health Scotland, Edinburgh, UK

## Abstract

**Background:**

The SHARP study was set up to evaluate the short (1 year) and longer-term (2 year) effects on health and wellbeing of providing new social housing to tenants. This paper presents the study background, the design and methods, and the findings at one year.

**Methods:**

Data were collected from social tenants who were rehoused into a new, general-purpose socially-rented home developed and let by a Scottish Registered Social Landlord (the "Intervention" group). These data were collected at three points in time: before moving (Wave 1), one year after moving (Wave 2) and two years after moving (Wave 3). Data were collected from a Comparison group using the same methods at Baseline (Wave 1) and after two years of follow-up (Wave 3). Qualitative data were also collected by means of individual interviews. This paper presents the quantitative and qualitative findings at 1 year (after Wave 2).

**Results:**

339 Intervention group interviews and 392 Comparison group interviews were completed. One year after moving to a new home there was a significant reduction in the proportion of Intervention group respondents reporting problems with the home, such as damp and noise. There was also a significant increase in neighbourhood satisfaction compared with Baseline (χ^2 ^= 35.51, p < 0.0001). Many aspects of the neighbourhood improved significantly, including antisocial behaviour. In terms of environmental aspects and services the greatest improvements were in the general appearance of the area, the reputation of the area, litter and rubbish, and speeding traffic. However, lack of facilities for children/young people and lack of safe children's play areas remained a concern for tenants.

**Conclusion:**

This study found that self-reported health changed little in the first year after moving. Nonetheless, the quantitative and qualitative data point to improvements in the quality of housing and of the local environment, as well as in tenant satisfaction and other related outcomes. Further analyses will explore whether these effects are sustained, and whether differences in health outcomes emerge at 2 years compared with the Comparison group.

## Background

Housing and regeneration activities may provide a key opportunity for improving the health of the public, and reducing health inequalities. The importance of the urban environment for health has also been emphasised recently by the WHO Commission on the Social Determinants of Health[1] Though most research in the area is descriptive rather than evaluative, the evidence base on housing and regeneration goes back many decades, with the earliest evaluation studies dating from the 1930s[2] In previous evaluative studies housing improvement has been shown to lead to small improvements in self-reported physical and mental health[2] However, controlled study designs are rare. The most recent study in this field, a large randomised controlled trial from New Zealand, suggested that improving the indoor environment by insulating older houses increased indoor temperatures and improved occupants' health and wellbeing[3] Few studies have examined housing improvement in the context of area regeneration, and where the effects of urban regeneration have been investigated, health outcomes have rarely been examined[4] It was against this background that SHARP (Scottish Housing Health and Regeneration Project) was conducted, with the aim of assessing the effects of new social housing and area regeneration on the health and wellbeing of tenants in the social-rented sector in Scotland. It adopted a controlled design, and aimed to identify the separate and additive effects of housing and area regeneration. At the time of the SHARP study, most public housing investment in Scotland related to the provision of new, general-needs housing, rather than the rehabilitation or improvement of older stock, as had been the case in earlier periods.

This paper outlines the background, aims, methods and short-term (1 year) findings of the SHARP Project.

### The intervention, design and outcomes

SHARP evaluated the effects of change in social housing -- specifically, the move to a newly built home, with the expected accompanying improvement in indoor conditions, such as greater warmth, eradication of damp and provision of greater space. However, there are other alterations to residents' circumstances which may accompany the move to a new house: for example, a change in housing provider and consequent changes in housing management practices, which include landlords' powers to deal with neighbourhood antisocial behaviour. Also, in getting a new house, people may also acquire a different local environment in terms of its physical qualities, the provision of services and facilities and the level of community activity and support[5] The main physical intervention being evaluated in the SHARP study therefore included changes not only in housing circumstances but also in neighbourhoods. It was not possible for the SHARP team to control the allocation of families to the new homes. We therefore identified an Intervention group (who moved into new social housing) and a matched Comparison group, and health and other outcomes were followed up in both groups over two years.

Although health outcomes were of primary importance, SHARP also aimed to evaluate the wider effects of new housing provision and to explore some of the mechanisms by which health is affected -- in particular, how housing and other types of neighbourhood change interact. We therefore collected data on the individual and area-level outcomes listed in Table 1.

**Table 1:** SHARP health and wellbeing outcomes

*Housing and area outcomes*

The home environment (including warmth, damp, mould, space and privacy; The affordability of rent and utility payments
*Neighbourhood outcomes*

Satisfaction with the area; Problems with the area; Friendliness of local people; Antisocial behaviour; Physical environment; Participation in local activities; Relationships with others in the local community (including social capital); Sense of "belonging" to the community; Use of local services and amenities

*General physical and mental health and wellbeing*

Long-standing illness (LSI) and common symptoms; Respiratory conditions; Health behaviours; Child health and school attendance; Health-related quality of life (SF-36);[19-21]; Accidents in and outside the home; The use of health and community services, such as GP consultations, hospital visits (emergency, out-patients and admittances) and use of medications

*Other psychosocial outcomes*

Sense of control over circumstances; Social participation, including social interactions with family, friends and neighbours; Sense of community; Perceptions of safety; Social capital; Psychosocial benefits of the home (e.g., privacy, identity, status)

### Context: Health improvement in regeneration areas

Housing investment increasingly occurs within a wider context of regeneration programmes. In Scotland, nearly 40% of the new-build housing in 1999/2000 (when SHARP was planned) were in urban regeneration areas, called Social Inclusion Partnerships (SIPs). This provided a rationale for investigating housing investment in the wider regeneration context.

SHARP therefore aimed to examine:

• The extent to which rehousing into a new socially rented dwelling delivers improvements (or indeed deteriorations) for occupants in terms of housing conditions, neighbourhood conditions, housing management performance and sense of community;

• Whether these improvements are associated with changes in a person's neighbourhood and landlord as well as rehousing itself; and

• To what extent people who are rehoused experience changes in their physical health, health behaviours and mental health and wellbeing, whether these health changes are sustained over time, and how they relate to changes in residential circumstances.

## Methods

A feasibility study in preparation for the full study was conducted in 2000. This piloted the survey instruments and tested practical aspects of the study. In the SHARP study itself, data were collected from social tenants who were rehoused into a new, general-purpose socially rented home developed and let by a Registered Social Landlord (the Intervention group)[6] These data were collected on three occasions: before moving (Wave 1, by face-to-face interview), one year after moving (Wave 2, by means of a postal survey) and two years after moving (Wave 3, by face-to-face interview). Data were collected from a Comparison group using the same methods at Baseline (Wave 1) and after two years of follow-up (Wave 3). Wave 1 versus Wave 2 comparisons (i.e., the short-term effects of the intervention) are reported here.

The Intervention and Comparison groups were matched at Baseline by housing tenure, area and a three-category household type (family households, with children under the age of sixteen years; older households, where the respondent and adult members of the household were of pensionable age; and adult households, with a combination of relationships, including parents with children at least 16 years of age, people unrelated to one another and couples). These categories were created in order to describe the broad range of adult households in the intervention group and were used to assist in recruiting the comparison group.

We asked respondents for permission to access routine NHS data on inpatient admissions. Approximately 90% of each group in the study gave this consent. These data describe all non-obstetric and non-psychiatric discharges and lengths of stay.

### Eligibility & Recruitment

The Wave 1 Intervention sample represents around 10% of the annual national output of general-purpose social-rented housing within the RSL sector in Scotland. Eligibility criteria for the intervention varies between housing providers and may include such issues as family size, health status and aspects of the accommodation. For example, an RSL may prioritise a particular family for a new home because the tenant has health problems. However, although health is often a consideration in allocating social housing, this is not always the case and housing needs and social factors are also very important.

Whilst RSLs can establish their own housing allocation criteria (i.e., eligibility and prioritisation rules) they must do this within a regulatory framework operated on behalf of the Scottish Government. Thus, the Scottish Housing Regulator's *'Performance Standards' *against which all RSLs are judged suitable for public funding says two things about access to social housing: first, there should be 'fair and open access' to the housing list for everyone (i.e., no exclusion criteria plus simple access routes); and, second, houses should be let in ways which 'give reasonable preference to those in greatest housing need; makes best use of available stock; maximises choice; and helps to sustain communities.'[7] The first of these lettings criteria -- 'greatest housing need' -- probably weighs most heavily in practice.

RSL concerns about data confidentiality meant that we were not able to approach prospective tenants directly to participate in the study. Instead, tenants were first contacted by the RSL with information about the study and were able to opt into the study. The SHARP research team supplied all the materials for this process. Recruitment was discussed with potential tenants once they had verbally accepted an offer of tenancy from the landlord. Whilst this gave a good chance of successful recruitment (since the future landlord was doing the asking), it also meant that we did not have detailed information on refusals, as RSLs could not supply this to us.

### Survey methods

At Wave 1, tenants who were about to move house were interviewed approximately 1-2 weeks before they moved into their new home, between May 2002 and April 2004. Rural and urban areas were both included in the survey, and approximately equal numbers of the households were in SIP and non-SIP areas.

At Wave 2, one year after moving, a postal survey was conducted among Intervention group respondents. This included a sub-set of questions from the Wave 1 interview schedule. First, general health, vitality and mental health were assessed using a commonly used health-related quality of life measure, the SF-36[8] Second, respondents reported on their experience of common symptoms in the previous month. Finally, the psychological benefits resulting from living in the dwelling were assessed. Of the completed questionnaires returned at Wave 2, most (81%) were returned by post and the remainder were collected in person by interviewers.

For Wave 3, face-to-face interviews were carried out again approximately two years after the Intervention group households had moved. The Comparison group were also re-interviewed. These interviews were conducted between May 2004 and April 2006 and as near as possible to the 12-month anniversary of the respondents' move to their new accommodation, although this was not always possible.

### Statistical analysis

We compared the proportions reporting particular outcomes at Baseline and follow-up using t-tests or chi-squared tests to compare percentages. In some cases we grouped the responses to particular questions into two categories in order to compare the percentage of respondents reporting a particular problem (or in some analyses, a "serious" problem) before and after the intervention. ANOVA/t-test or the Mann Whitney U-test were used to compare means and medians as appropriate.

### Qualitative data collection

Twenty-eight individual qualitative interviews (18 women and 10 men) were conducted with the Intervention group 1-3 years after the move, and a further 22 interviews were conducted with a different sub-sample at 3-5 years (18 women, 10 men -- not reported here). The qualitative research component aimed to focus on the impacts of housing and neighbourhood change on the lives of respondents; to identify issues to pursue in the final quantitative analysis; and to identify those aspects tenants had cited as being of greatest importance in the housing-health-regeneration nexus. Thirteen (46%) interviews were conducted with women in a 'family' household, six (21%) with respondents living in an 'adult' household and 9 (32%) in 'older person' households. The interview questions covered five broad themes, within which the interview data were grouped for the purposes of analysis: (i) general questions about the location where the respondent lived, and recent changes; (ii) the respondent's relationship with their new home, including the process of moving home, comparisons between the old and new houses; (iii) relationships with the population in the area; (iv) health of the respondent and, if relevant, that of others in the household, and health-related behaviours; and (iv) respondent's attachment to the local area.

Ethical approval for SHARP was granted by the University of Glasgow Ethics Committee, and the Multi-Centre Ethics Committee for Scotland.

## Results

### Achieved samples

#### Wave 1: Pre-Rehousing

46% of those approached agreed to participate, resulting in 339 Intervention group interviews. Locations in and around Glasgow accounted for 49.1% and 51.9% of the total number of Intervention and Comparison group interviews respectively, though this distribution reflects the relative concentration of RSLs in Scotland. It is worth noting that SHARP is the first housing intervention study in Scotland with national coverage: the sample involved 57 housing developments built by 45 RSLs across 21 local authority areas, thus diminishing any site-specific effects upon the outcomes.

### Comparability of Intervention and Comparison groups at Wave 1

Women were the most frequent respondents (73.0% overall) (Table 2). The Intervention group were younger (43 *vs*. 50 years, p < 0.001), perhaps reflecting less willingness to move with age. Comparing tenure types, the comparison group were more likely to be in council/housing association property. About 62% of the Intervention group households were living in family households, compared with 45% in the Comparison group; similarly, there was a significantly lower proportion of older households in the Intervention group (10% *vs*. 22%). About 2% of both groups were from non-white ethnic populations, broadly reflecting the ethnic make-up of Scotland as a whole. The Intervention group were more likely to possess higher educational qualification (57% *vs*. 41.5%; χ^2 ^= 12.7; p < 0.001), and were also more likely to be working. Overall weekly income was similar in the two groups.

**Table 2:** Description of Intervention and Comparison groups at Baseline (Wave 1)

	Intervention group n (%)	Comparison group n (%)	Chi-squared
**Sex**			
*Male*	77 (23.1)	117 (30.4)	χ^2 ^= 4.52, p = 0.034
*Female*	257 (76.9)	268 (69.6)	
**Age**(years)			
*Mean*	43.2	49.7	F = 29.8, p < 0.001; U = 46225.5, p < 0.001
*Median*	41	47.5	
**Tenure**			
*Council/Housing association*	239 (71.6)	373 (97.1)	χ^2 ^= 90.6, p < 0.001
*Other*	95 (28.4)	11 (2.9)	
**Marital status**			
*Married or with partner*	117 (35)	155 (39.9)	χ^2 ^= 18.4,
*Other*	217 (65)	234 (60.1)	p < 0.001
**Family unit type**			
*Family*	206 (61.7)	174 (44.7)	χ^2 ^= 6.6,
*Adult*	96 (28.7)	133 (34.2	p < 0.001
*Older*	32 (9.6)	82 (21.1	
**Ethnicity**			
*White*	327 (97.9)	382 (98.2)	χ^2 ^= 14.2, p = 0.028
**Education (highest level)**			
*School leaving certificate*	49 (18.7)	62 (21.8)	χ^2 ^= 0.82, p = 0.364
			χ^2 ^= 8.63, p = 0.003
*O Grade/Standard Grade/GCSE/CSE/Senior Certificate*	112 (41.7)	87 (30.6)	
*GSVQ foundation or intermediate/SVQ Level 1 or 2, SCOTVEC module or equivalent*			χ^2 ^= 3.47, p = 0.062
*Higher Grade/CSYS/*	44 (16.8)	32 (11.3)	
*A level/Advanced Senior Certificate or equivalent*			
			χ^2 ^= 2.31, p = 0.129
*GSVQ Advanced/SVQ Level 3/ONC/OND/SCOTVEC National Diploma or equivalent*			
*City & Guilds*	39 (14.9)	39 (10.6)	χ^2 ^= 5.39, p = 0.020
*HNC/HND/SVQ Level 4 or 5 or equivalent*			
	24 (9.2)	12 (4.2)	χ^2 ^= 0.586, p = 0.444
*First or higher degree*			
			χ^2 ^= 6.83, p = 0.009
*Professional qualification (e.g., teaching, accountancy)*			χ^2 ^= 0.04, p = 0.847
	20 (7.6)	17 (6.0)	
*Other qualification*			χ^2 ^= 0.02, p = 0.898
	26 (9.9)	12 (4.2)	χ^2 ^< 0.001, p = 0.986
*None*			
			χ^2 ^= 5.223, p = 0.022
	11 (4.2)	11 (3.9)	
	5 (1.9)	5 (1.8)	
	13 (5.0)	14 (4.9)	
	78 (29.8)	111 (39.1)	
**Employment status**			
Currently working*	126 (37.7)	111 (28.5)	* χ^2 ^= 6.48, p = 0.01
*Unemployed*	36 (11)	44 (11.3)	
*Sick/disabled*	61 (18.7)	67 (17.3)	
*Retired*	38 (11.6)	101 (26)	
**Median income/week**	£178.80	£175.12	U = 15245.5, p = 0.74
In SIP*	170 (50.9)	183 (47.2)	χ^2 ^= 0.93,
			p = 0.335
**Median number of people living in household**	3	2	U = 54681.5,
			p < 0.001
**Housing type**			
*Detached/semi-detached/terraced house*			
*Tenement or flat*	94 (28.2)	186 (47.8)	χ^2 ^= 1.31,
*Other*	230 (69.1)	184 (47.3)	p < 0.001
	9 (2.7)	19 (95.1)	
**General self-reported health:**			
*Good, very good or excellent*			
	197 (58)	221 (56.8)	χ^2 ^= 1.05,
			p = 0.9
**Long-standing illness**	185 (55.4)	242 (62.2)	χ^2 ^= 3.2,
			p = 0.07

* Regeneration area

χ^2 ^= Chi-squared value; F = F statistic for comparison of means; U = Mann Whitney U

### Geographical distribution: Urban/Rural, and SIP/non-SIP classification

The Intervention and Comparison groups mostly consisted of households in urban areas (almost 80%). More than half of the households were situated in large urban areas. The distribution of households was similar in the two groups (χ^2 ^= 7.7, p = 0.16). Of the 279 Intervention group respondents for whom SIP status could be determined, 58% were located in SIPs, whilst 46% of the Comparison group population lived in a SIP area.

### Accommodation characteristics

The median household size for the Intervention group was slightly greater (3 *vs*. 2 persons; p < 0.001). At Wave 1, over two-thirds (69.1%) of the Intervention group lived in flats or tenements (in Scotland, a tenement usually refers to a low-rise block of flats with a common stairwell). The Comparison group sample at Wave 1 were equally split between houses (47.8%) and flats/tenements (47.3%). This pattern might be expected since tenement-dwellers are more likely to be rehoused.

### Health

There was no difference at Baseline in self-reported health between the Intervention and Comparison groups at Wave 1 (59.0% *vs*. 56.7% respectively reporting general health as good or better; χ^2 ^= 0.2, p = 0.70). Men in the Comparison group were more likely to report a long-standing illness (72.4% *vs*. 57.1%, p = 0.03). There was no difference for women (58.6% *vs*. 55.5%, p = 0.48).

We carried out bivariate analyses to determine whether any of the other characteristics which differed significantly between the two study groups were related to a change in self-reported health within the Intervention group between Waves, and thus would be likely to act as confounders in subsequent analyses. There was no significant relationship between change in self-reported health (in two categories: "excellent", "very good" or "good", versus "fair" or "poor") between Waves, and any other variable: household type (χ^2 ^= 4.5, p = 0.35), sex (χ^2 ^= 1.4, p = 0.5), marital status (χ^2 ^= 6.1, p = 0.41), employment status (χ^2 ^= 3.3, p = 0.20), tenure (χ^2 ^= 19.5, p = 0.15); household size (χ^2 ^= 10.7, p = 0.71), ethnicity (χ^2 ^= 4.5, p = 0.61) or housing type (χ^2 ^= 24.9, p = 0.41).

### Changes between Wave 1 and Wave 2: Postal Survey (Intervention group only)

Respondents had lived in their new house for an average of 12.8 months before participating in the Wave 2 postal survey. A response rate of 83.8% (280/334; 79.3% female, compared with 76.9% female at Wave 1) was achieved. The age structure of the two groups was also very similar, with those aged 30-39 years making up the largest group of respondents. There was no change in the distribution of household types (data not shown). Non-responders at Wave 2 were more likely to be male (24.7% of men did not respond at Wave 2, compared to 13.6% of women; χ^2 ^= 4.56; p = 0.03). Those aged under 50 and over 70 were less likely to respond (25% of those aged under 30; 16.7% of those aged 30-49; 6.4% of those aged 50-69; and 25% of those aged 70 or over were non-responders; χ^2 ^= 10.7; p = 0.01). There was no statistically significant association between non-response and either education, or working status (data not shown).

In general, the distribution of respondents across postal areas was similar at Baseline and after 1 year. In total, one-in-eight (12.4%) of the Intervention group respondents moved between settlement categories (i.e., Urban/Rural) between the Baseline interviews and the Postal Survey, though the vast majority (87.6%) stayed in the same type of urban/rural classification. There was no significant change in the SIP status of respondents as a result of their relocation to new accommodation. (Wave 1: 49.1% *vs. *Wave 2: 51.9%).

### Short-term effects of housing change at 1 year (Wave 1 vs. Wave 2)

#### Housing and residential change

The majority of Intervention group respondents (54.7%) retained the same landlord after the move. Just over half of households considered themselves still to be living within the same neighbourhood (53.2%). The rest perceived themselves to have moved to a new neighbourhood (46.8%). For the Intervention group, rehousing often resulted in a change in the type of dwelling occupied: before moving, only 28% lived in a house, compared with 63% afterwards.

#### Problems with the home

Before the move to a new home a substantial proportion of respondents reported problems with damp, warmth in winter, privacy, lack of space, and noise; over one-third of tenants had reported that at least one of these was currently a problem. After moving there were significant reductions in the proportion of respondents reporting such problems -- this was true for 10 out of 11 housing problems investigated. The greatest improvements concerned dampness and keeping warm in winter, with the problem of damp largely being eliminated (Table 3). Although difficulties with keeping warm in winter also reduced substantially (a reduction from 41.4% reporting a problem to 14.5%; p < 0.0001), around one-in-seven respondents still reported this as a problem despite having moved into a new home built to current standards.

**Table 3:** Change in prevalence of problems with the home

	Number (%) claiming this is a problem		
	
Problem	Baseline Survey (Wave 1)	Postal Survey (Wave 2)	Percentage decrease (n), t statistics
Damp	94 (36.0)	8 (3.1)	32.9% (261) (t = 9.96; p < 0.0001)

Keeping home warm in winter	110 (41.4)	38 (14.5)	26.9% (266) (t = 6.29; p < 0.0001)

Not enough privacy	97 (36.9)	59 (22.4)	14.5% (263) (t = 3.11; p = 0.02)

Problems getting in or out of home	55 (22.7)	24 (9.7)	13.0% (243) (t = 3.66; p = 0.0003)

Smells and fumes	52 (21.4)	21 (8.6)	12.8% (243) (t = 3.75; p = 0.0002)

Noise from other household members	59 (26.8)	31 (14.2)	12.6% (220) (t = 2.98; p = 0.003)

Rooms too small	113 (42.0)	84 (31.2)	10.8% (269) (t = 2.09; p = 0.04)

Accidents outside the home	49 (18.4)	25 (9.4)	9.0% (266) (t = 2.83; p = 0.005)

Noise from neighbours	104 (38.7)	81 (30.1)	8.6% (269) (t = 1.71; p = 0.09)

Accidents inside the home	35 (13.1)	14 (5.2)	7.9% (267) (t = 3.07; p = 0.002)

Rooms too large	6 (2.4)	2 (0.8)	1.6% (254) (t = 1.43; p = 0.15)

Variation in totals is largely due to errors in completion of the postal questionnaire

Most interviewees had moved to a property that had a front and back door opening onto their own front and back gardens: the proportion of the Intervention group who had sole use of a garden nearly doubled from 41.0% to 78.2%. Nevertheless, a significant proportion of interviewees still reported problems with noise from other household members (14.2%), noise from neighbours (30.1%) and a lack of privacy (22.4%), even though these had all reduced significantly compared with the situation in their previous homes.

#### Affordability

A number of studies have suggested that housing change may be accompanied by decreases in affordability, and that this may have health consequences because it reduces expenditure on food[9] In the SHARP study respondents were asked how frequently they had difficulty paying a range of household charges. There were increases in the proportion of respondents finding it difficult to afford council tax, fuel bills, food, rent and telephone bills over the year, these were more than balanced by the large majority of respondents who reported that there had been no change, or even that it had become easier (Additional File 1). The main expense which most frequently proved difficult for people to meet was housing repairs, with 42.1% reporting that this had become more difficult after moving. This may be due to the 'snagging' problems often experienced early on in a new home, and the wish to make small changes to the property. We might therefore expect to see repairs becoming less of an issue at Wave 3.

#### Neighbourhood change, antisocial behaviour and friendliness

There was a significant increase in neighbourhood satisfaction compared with Baseline, with the proportion reporting that this was a "very good" or "fairly good" place to live, increasing from 64.1% before moving, to 79% after the move (χ^2 ^= 35.5, p < 0.0001). People who reported that they had moved from their original neighbourhood (47% of the sample) were significantly more likely to rate their new area as "fairly good" or "very good" (87.4%) compared with those who had moved within the same neighbourhood (71.7%) (χ^2 ^= 8.8, p = 0.003).

Twenty-three specific aspects of the neighbourhood were asked about at both Waves 1 and 2 (Table 4). Fifteen of these aspects improved over time (i.e., problems were significantly reduced in the perception of respondents). The greatest improvements (i.e., absolute reductions in percentages) tended to be in antisocial behaviour: the percentage of tenants reporting that vandalism/graffiti, drug dealing and drug taking, and alcohol consumption in public, were serious problems all decreased by more than 20% in absolute terms between Wave 1 and Wave 2. Significant reductions in problems with security and police response were also noted.

**Table 4:** Change in prevalence of neighbourhood problems

Problem	Percentage of households claiming this is a problem (either a minor or serious problem). *In italics: % reporting serious problem % (n)*		
	**Baseline Survey (Wave 1)**	**Postal Survey (Wave 2)**	**Percentage change (n)**

*Antisocial Behaviours* Vandalism/graffiti	63.6 *(38.0% 127/332)*	47.6 *(10.2% 34/267)*	-16.0% Δ = -27.8 *(t = 7.75;p < 0.0001)*

Drug dealing/taking	61.8 *(37.7%; 126/301)*	50.8 *(17.1%; 57/266)*	-11.0% Δ = *-20.6; t = 5.45; p < 0.0001*

Assaults or mugging	40.7 *(17.7%; 59/317)*	32.6 *(5.4%; 18/267)*	-8.1% Δ = *-8.1; t = 4.54; p < 0.0001*

People drinking alcohol in public places	56.1 *(33.2%; 111/328)*	49.8 *(12.3%; 41/269)*	-6.3% Δ = *-20.9; t = 5.97; p < 0.0001*

Burglaries	33.0 *(9.6%; 31/318)*	27.8 *(2.1%; 7/266)*	-5.2% Δ = *-5.2; t = 3.75; p = 0.0002*

People hanging round	45.6 *(31.7%; 106/333)*	54.6 *(15.3%; 51/271)*	+1.0% Δ = *-16.4; t = 4.67; p < 0.0001*

The people round here	*31.3 (9.6%; 32/332)*	30.9 *(3.3%; 11/269)*	-0.4% Δ = *-6.6; t = 3.06; p = 0.002*

Domestic abuse	19.4 *(3.6%; 12/263)*	19.5 *(2.7%; 9/256)*	+0.1% Δ = *-0.9; t = 0.59; p = 0.56*

Disturbance by children/youngsters	55.3 *(23.1%; 77/333)*	61.6 *(12.9%; 43/271)*	+6.3% Δ = *-10.2%; t = 3.21; p = 0.001*

Nuisance from dogs	40.3 *(17.1%; 57/330)*	49.6 *(14.4%; 48/272)*	+9.3% Δ = *-2.7%; t = 0.90; p = 0.37*

*Environmental Aspects & Services*			

General appearance of area	51.8 *(24.3%; 81/332)*	30.8 *(4.2%; 14/273)*	-21.0% Δ = *-21%; t = 6.85; p < 0.0001*

Uneven/dangerous pavements	50.3 *(21.6; 72/332)*	36.1 *(9.6%; 32/269)*	-14.2% Δ = *12%; t = 3.97; p = 0.0001*

Level of police presence/response speed	60.5 *(36.8; 123/299)*	49.4 *(15.6%; 52/267)*	-11.1% Δ = *-11.1%; t = 5.69; p < 0.0001*

Adequate street lighting	22.1 *(6%; 20/331)*	15.1 *(3.6%; 12/272)*	-7.0% Δ = *-7%; t = 2.18; p = 0.03*

Reputation of area	52.3 *(29.3%; 98/331)*	43.2 *(12.3%; 41/271)*	-9.1% Δ = *-17%; t = 5.04; p < 0.0001*

Litter and rubbish	60.1 *(26.9%; 90/333)*	53.6 *(9.9%; 33/272)*	-6.5% Δ = *-17%; t = 5.28; p < 0.0001*

Air quality/pollution	29.1 *(9.3%; 31/327)*	23.5 *(3.6%; 12/272)*	-5.6% Δ = *-5.6%; t = 2.78; p = 0.006*

Public transport services	35.6 *(16.8%; 56/320)*	31.4 *(10.5%; 35/271)*	-4.2% Δ = *-4.2%; t = 2.21; p = 0.03*

Security levels of houses, closes, courts and gardens	40.3 *19.2% (64/330)*	37.1 *7.2% (24/267)*	-3.2% Δ = *-12%; t = 4.22; p < 0.0001*

Facilities for children/young people	86.7 *(63.5%; 212/309)*	84.3 *(41.9%; 140/267)*	-2.4% Δ = *-2.4%; t = 5.18; p < 0.0001*

Speeding traffic/amount of traffic	60.7 *(30.8%; 103/333)*	60.4 *(13.8%; 46/270)*	-0.3% Δ = *-17%; t = 4.92; p < 0.0001*

Safe children's play areas	65.4 *(42.2%; 141/315)*	65.1 *(27.5%; 92/269)*	-0.3% Δ = *-14.7%; t = 3.7; p = 0.0002*

Noise e.g. factories, traffic, shouting	40.2 *(12.9%; 43/333)*	46.7 *(9.6%; 32/272)*	+6.5% Δ = *-3.3%; t = 1.27; p = 0.21*

Variation in totals is largely due to errors in completion of the postal questionnaire

In terms of environmental aspects and services the greatest improvements were the general appearance of the area (an absolute reduction of 21% in the reporting of a problem), and the condition of pavements (a reduction in the reporting of problems of 14%). The percentage of those reporting that speeding traffic was a serious problem reduced by 17%. However lack of facilities for children/young people, lack of safe children's play areas and speeding traffic all remained sources of concern at both times, with three-in-five or more identifying these as local problems after rehousing.

At Baseline and 1 year respondents were asked about the friendliness of the local population. The percentage reporting that people were "quite friendly" or "very friendly" changed little over time and did not differ between those who had moved into a new neighbourhood and those who had remained in the same area. (χ^2 ^= 5.9, 4 d.f., p = 0.21).

Further post-hoc analyses were conducted comparing the experiences of those who moved home and neighbourhood with those who moved home but stayed in the same neighbourhood. These are provided in Additional File 2 (see last column of table). In brief, there are few apparent differences between those who moved neighbourhood and those who did not, and little evidence of a consistent pattern where differences were identified. The reported reduction in vandalism/graffiti, and reported improvement in the general appearance of the area were greater in those who stayed in the same neighbourhood (>10% difference in absolute terms). Those who moved area were less likely to report that burglaries were a serious problem (an absolute difference of over 15.1%) and also less likely to report that lack of facilities for children/young people were a problem (an absolute difference of 20.9%).

### Changes in self-reported health and wellbeing

Self-reported health improved at Wave 2. Whilst 17.7% of respondents in the Baseline survey had rated their health as "poor", this dropped to 10.5% by one year later (t = 2.5; p = 0.01). Although the improvement is statistically significant, there was no association between change (increase, same, or decrease) in the number of housing problems reported, and change in health (worse, better, the same) (χ^2 ^= 0.16; p = 0.13). The mean SF-36 mental health scores changed little over time, from 58.3 at Baseline, to 59.2 one year later (p = 0.51). In contrast to mental health, there was a significant increase in SF-36 vitality scores (means of 42.3 and 51.5, respectively; p < 0.001), low SF-36 vitality scores being an indicator of fatigue/exhaustion.

Overall respondents cited slightly fewer symptoms after they moved house than they had when interviewed one year before, though the difference was not statistically significant (means at Baseline and Wave 2: 3.27 (95% CI: 3.03-3.51), 3.09 (95% CI: 2.81-3.37). However, there were declines in the percentages of individuals who reported suffering from specific common symptoms (Table 5), although using a conservative cut-off probability (p = 0.01), the only significant reduction was in the prevalence of people reporting difficulty sleeping (down 17.4%, p < 0.0001). This was not due to reduction in noise, however, as the association between change in difficulty sleeping and change in problems with noise was not significant (χ^2 ^= 1.7; p = 0.79).

**Table 5:** Percentage reporting particular symptoms in past month

	Percentage (number) claiming this is a problem		
**Symptom**	**Baseline Survey (Wave 1)**	**Postal Survey (Wave 2)**	**Change %**

Hay fever	14.7 (334)	11.4 (259)	Δ = -3.3 (t = 1.18; p = 0.24)

Difficulty sleeping	63.2 (333)	45.8 (263)	Δ = -17.4 (t = 4.24; p < 0.0001)

Indigestion/stomach trouble	37.7 (334)	32.9 (260)	Δ = -4.8 (t = 1.21; p = 0.23)

Eye trouble	25.7 (334)	18.3 (255)	Δ = -7.4 (t = 2.13; p = 0.03)

Painful joints	50.3 (334)	41.6 (265)	Δ = -8.7 (t = 2.12; p = 0.03)

Palpitations/breathlessness	39.2 (334)	31.4 (265)	Δ = -7.8 (t = 1.98; p = 0.048)

Ear trouble	21.0 (334)	15.3 (259)	Δ = -5.7 (t = 1.77; p = 0.08)

Sinus trouble/catarrh	29.6 (334)	24.9 (261)	Δ = -4.7 (t = 1.27; p = 0.20)

Persistent cough	24.3 (334)	22.2 (261)	Δ = -2.1 (t = 0.6; p = 0.55)

Faints/dizziness	21 (334)	15.3 (257)	Δ = -5.7 (t = 1.77; p = 0.08)

Finally, tenants were asked about a range of psychosocial benefits from the home using a previously developed scale[10,11] They reported significant improvements in most items, especially status or pride ("Most people would like a home like mine"), sense of personal progress ("My home makes me feel I am doing well in life") and identity ("My home expresses my personality and values") (Table 6).

**Table 6:** Psychosocial benefits from the new home

Statement	Percentage (number) claiming this is a problem		
	**Baseline Survey: % agreeing (n)**	**Postal survey (Wave 2) % agreeing (n)**	**Percentage change (absolute difference)**

My home makes me feel like I'm doing well in my life	30.6 (102)	65.1 (179)	+34.5 (t = 8.48; p < 0.0001)

My home expresses my personality and values	43.1 (143)	72.8 (201)	+29.7 (t = 7.33; p p < 0.0001)

I feel in control of my home	57.5 (191)	79.4 (216)	+21.9 (t = 5.71; p p < 0.0001)

I can do what I want, when I want in my home	61.3 (204)	81.2 (224)	+19.9 (t = 5.35; p p < 0.0001)

I can get away from it all in my home	56.3 (187)	75.7 (206)	+19.4 (t = 4.97; p p < 0.0001)

I feel I have privacy in my home	67.9 (226)	83.2 (227)	+15.3 (t = 4.31; p p < 0.0001)

My home feels safe	75.9 (252)	87.5 (239)	+11.6 (t = 3.63; p p < 0.0001)

My life has a sense of routine	79.2 (262)	78.5 (215)	-0.7 (t = 0.21; p = 0.58)

I worry about losing my home	21.0 (70)	26.6 (71)	+5.6 (t = 1.61; p = 0.95)

#### Qualitative data on housing change

Improvements were also clear from the qualitative data we collected, which suggest that people experienced improvements in wellbeing, and in particular a reduction in stress, both within the family unit and in relations with neighbours. This appears to be related to having greater space, privacy and a safer, more peaceful local environment.

Many of the respondents also reported that they had more time to relax in their new house; they felt less stressed, were happier and felt secure. For example, this respondent spoke about her health and that of her son:

'*I'd say our health has improved... now here we are just over a year on and we are a lot more content, we are a lot more settled, and we are a lot more happier because I feel like my head's back and I'm not needing anti-depressants and things, you know. I'm feeling a lot more positive...."*

For many respondents, the greater space and privacy in their new homes was the greatest benefit of the move. In particular it meant that children had separate rooms rather than having to share with a sibling. For one interviewee, this meant "*a happier house*". For another respondent, a larger kitchen now meant enough room for a tumble drier and washing machine, and for another it meant that the family did not have to eat from their laps in the sitting room.

One frequently mentioned benefit of the new homes was the greater space resulting from having a garden. Some parents commented that with more garden access their children were getting out and about more, while gardens also provided space in which they could sit out and relax, or choose to socialise with their neighbours.

Many of those interviewed stated that there had been significant improvements in health, either for children in the household or for themselves. For example:

"...when he was younger he was quite wheezy for a while and it always seemed to be in the winter when the heating was on... they actually thought that he was going to develop asthma, but since being over here, he's been fine."

Another interviewee said:

"*...they don't get quite so many colds as they used to and because we lived in a damp house when [son] was a baby it affected his chest [...] his chest is not quite so bad because we're living in a drier atmosphere."*

One man with respiratory problems thought his health had improved since moving in:

"*Well, I've not been admitted to the hospital since I've come round here... [I'm] ... a lot healthier than what I used to be. As I say, I've got more freedom of movement because I'm getting into fresh air a lot more, which is probably helping as well*."

Another participant stated that his wife had also been able to come off anti-depressants, whose use the couple felt was related to the problems they had been experiencing with drug addicts using the close (that is, the stairwell or common area) of their previous home. The change in the health of both of them was, the husband thought, '*immediate'*. A few respondents also reported that they were thinking about making changes to health-related behaviours, such as smoking.

There were also many who identified no change in their health, some of whom simply thought that they were getting older and that any change in wellbeing was simply the result of this process. Similarly, others noted no change in smoking:

"*Just the same. I know, I'm not meant to be doing it but I do it."*

Another interviewee reported that as well as continuing to smoke, her diet may have become more unhealthy:

"I smoke more now than I ever do, but that's just me, I've always been a heavy smoker ... I've got a wee bit more money than I did over there [...] there're definitely always more crisps in the cupboard, more sweeties in the cupboard..."

## Discussion

This study found that rehousing resulted in dramatic improvements in two major aspects of housing quality: damp and cold. Other aspects also improved, including noise, access and privacy, as might be expected, given that over a third of this group moved from flats (such as traditional tenements) to houses. There was also a significant increase in neighbourhood satisfaction, perhaps due to the improvements in general appearance and reductions in antisocial behaviour. There were only slight changes in self-reported health at 1 year, and even these should be considered in the light of the uncontrolled nature of the data at 1 year, and the fact that they are self-reported. The additional analyses we conducted comparing those who moved neighbourhood and those who stayed in the same neighbourhood did not suggest any consistent pattern. The largest difference observed was in relation to facilities for children and young people, in which a greater improvement was reported by those who moved area. This may reflect the possibility that neighbourhood change is necessary when there are wider improvements to an area, over and above any housing improvements. Such wider improvements may then include new or better facilities.

From these short-term findings, however, it appears that significant changes in health in the short-term are not likely to result from housing and neighbourhood improvement, even where there are substantial improvements in dwelling and neighbourhood conditions (as in this case). The relationship between any changes in mental and physical health and the home and local environment in the longer term require further analyses using the data from the comparison group at 2 years of follow-up.

### Previous studies of housing and health

The findings need to be put in the context of previous studies of housing improvement and of regeneration. Although the association between poor housing and poorer health has been well established from observational studies, the evidence from evaluations of the effects of housing improvement is somewhat weaker. However, general health has been found to improve following improvements to heating or insulation in controlled studies conducted in New Zealand (an RCT) and Scotland[3,12] A systematic review has also found that while improvements have been reported in overall self-reported physical and mental health, as well as reductions in symptoms, it was not possible to specify the nature and size of the health gain that may result from a specific housing improvement[13]

There have also been evaluations of the effects of housing change on specific symptoms or health conditions. Two UK studies which have assessed respiratory health following housing improvement have found evidence of significant improvements[14,15]. We also found a reduction in problems of breathlessness (-7.8%, p = 0.048), but the largest and most significant reduction in the SHARP study was in problems with sleeping (-17.4%, p = < 0.0001), which may be related to the less stressful environment reported by respondents in the in-depth interviews. With respect to mental health, this tended to improve in the majority of previous evaluative studies which examined this outcome,[11] but in the current study, significant changes in mental health following rehousing at 1 year were not detected.

### Financial strain

A small proportion of respondents in this study reported that rehousing had been followed by decreases in the affordability of some household bills or services; nevertheless, most found no change, or an improvement. One other UK study carried out in Liverpool reported a reduction in financial strain on the household at 1 year, resulting from improvements in energy efficiency following rehousing,[16] and an RCT conducted in New Zealand that evaluated the effects of retrofitting insulation found that energy efficiency increased, equating to the insulated households having 81% of the energy consumption of the comparison group after the intervention[3] It is worth bearing in mind here that the new properties occupied by social sector tenants in Scotland are also likely to have much better heating systems and to be more energy efficient than the post-war tenemental housing previously occupied by many participants, which were of notoriously poor construction. Thus, in the SHARP study, one year after rehousing, it was found that for 7 out of 9 household items, gains in affordability (i.e., reductions in the prevalence of payment difficulties) exceeded affordability losses. The main exception to this was housing repair costs (where increases in payment difficulties was the most common outcome), but this may well be a short-term consequence of moving to a newly constructed home with 'snagging' problems and the initial need to adapt the home to suit one's needs.

### Neighbourhood change

There is a considerable and growing literature on the association between characteristics of local neighbourhoods and health and wellbeing. In particular, perceptions of neighbourhood characteristics (such as neighbourhood quality and the neighbourhood problems as measured in the SHARP study) have been shown to be associated with self-rated health -- in particular, the effects on health of neighbourhood deprivation may be mediated by perceptions of the neighbourhood[17] Other studies have also shown associations between neighbourhood conditions and chronic health conditions and mental health[18]

Previous studies of housing-led neighbourhood renewal have reported changes in residents' perceptions of the local neighbourhood, and in the locality as a place to live, and two studies which examined the effects of housing improvement in the context of area regeneration also reported that residents' concerns about local crime were reduced[2] This was also found in the SHARP study and, indeed, the large reductions in the perception of antisocial behaviour as a serious problem are both significant and stronger than in the previous studies. Whether these improvements are sustainable in the longer term or reflect a short-term "honeymoon effect" will be determined in analyses of the 2-year outcome data.

### Strengths and limitations

These findings have several key limitations, the major one at this stage being the lack of a comparison group at this point. This limits the interpretation of the findings, although the changes with respect to the home and neighbourhood environment are large and undoubtedly real. However comparison group data are available at 2 years after the move, analysis of which will allow a clearer picture of the effects of housing and neighbourhood change to emerge.

The small study size also limits the power to detect small changes in health status and other outcomes. However this is an unusual study in that it involves prospective evaluations of the health and social outcomes of housing and neighbourhood renewal, and there are few such studies in the public health evidence base. It is also unusual in that it takes a holistic, multidisciplinary view of the outcomes of housing improvement -- including wider health and wellbeing and social outcomes. It is thus well placed to test some of the claims made about the potential for social housing and neighbourhood improvement not just to improve living conditions, but also to reduce fear of crime, improve social functioning (data were collected on this outcome at Waves 1 and Wave 3) and contribute to reducing health inequalities.

One final limitation relates to the fact that we were not able to approach tenants directly in order to draw our sample. Instead, tenants were first contacted by the Resident Social Landlord (RSL) and allowed to opt into the study. This meant that we did not have information on refusals and so the generalisability of the findings is unclear, though they are consistent with what we expected from the existing literature on the limited effects of housing improvement on mental health and wellbeing outcomes.

## Conclusion

Despite the limitations outlined above, the short-term findings from the SHARP study provide evidence that social housing renewal is accompanied by significant improvements in the internal housing environment and by large, positive changes in the local environment, and that these are reflected in psychosocial benefits to tenants. Although in the short term these benefits were not accompanied by significant changes in health, they are indicators of significant improvements in quality of life, as corroborated by the qualitative data.

The lack of significant benefits to health, while perhaps disappointing to those who advocate housing investment as a major tool for improving health and reducing health inequalities, are broadly consistent with other research to date[4,13] The reasons for these limited impacts, given the well-documented associations between poor housing and poor health, are less clear. We cannot discount the possibility that the effects of housing on population health in the 21st century may be modest in high-income countries. That is not to deny the clear historical evidence that poor physical housing conditions in the slums of the 19th and first half of the 20th century had a major, detrimental effect on health. However, housing standards may now be generally higher in high-income countries, to the extent that we may now be experiencing ceiling effects with respect to health improvement; that is, the housing being improved or renovated was not itself significantly health-damaging.

Our analyses of the final outcomes at 2 years will explore these issues further and will include an assessment of the effects of housing and neighbourhood change on the wider range of health and social outcomes in relation to the Comparison group. As well as exploring the effects of environmental change, further analyses will also explore the role of psychosocial factors (such as control, and support), which have been hypothesised to contribute to health and health inequalities[19]

## Competing interests

The authors declare that they have no competing interests.

## Authors' contributions

The study was originally conceived by AK and MP. AK, MP and CH contributed to the design of the study. PM carried out the analyses. All authors helped to draft and revise the manuscript, and read and approved the final version.

## Pre-publication history

The pre-publication history for this paper can be accessed here:

http://www.biomedcentral.com/1471-2458/9/415/prepub

## Supplementary Material

Additional file 1 **Figure 1: Change in affordability after moving home**. Stacked bar chart showing reported change in affordability of various items/utilities after moving home. Click here for file

Additional file 2 **Percentage of households claiming this is a minor or serious problem**. Table comparing the experiences of those who moved home and neighbourhood with those who moved home but stayed in the same neighbourhood. Click here for file
