# Supplementary material for: The SHARP study: a quantitative and qualitative evaluation of the short-term outcomes of housing and neighbourhood renewal
Source: BMC Public Health. 2009 Nov 17;9:415. doi: 10.1186/1471-2458-9-415 (PMC2784462; doi:10.1186/1471-2458-9-415)
Supplement: Additional file 2 — Percentage of households claiming this is a minor or serious problem. Table comparing the experiences of those who moved home and neighbourhood with those who moved home but stayed in the same neighbourhood. [file 1471-2458-9-415-S2.DOC]

|  | **Additional File 1: Percentage of households claiming this is a minor or serious problem. *In italics: serious problem only % (n/total)*** | | | | | |  |
| --- | --- | --- | --- | --- | --- | --- | --- |
|  | **New Home, Same Neighbourhood** | | | **New Home, Different Neighbourhood** | | |  |
| **Problem in the Neighbourhood** | **Baseline Survey** | **Postal Survey (1 year after)** | **Change %^a^** | **Baseline Survey** | **Postal Survey (1 year after)** | **Change %^a^** | **Absolute difference*** |
| *Antisocial Behaviours* |  |  |  |  |  |  |  |
| Vandalism/graffiti | 71.4 | 60.0 | -11.4 | 56.6 | 32.8 | -23.8 |  |
|  | *53.1 (78/147)* | *20.0 (29/145)* | *-33.1* | *23.3 (30/129)* | *4.1 (5/122)* | *-19.2* | 13.9 |
| Drug dealing/ taking | 71.3 | 64.1 | -7.2 | 50.4 | 34.7 | -15.7 |  |
|  | *50.7 (69/136)* | *31.0 (45/145)* | *-19.6* | *31.0 (35/113)* | *9.9 (12/121)* | *-21.1* | -1.5 |
| Assaults or mugging | 47.6 | 42.7 | -4.9 | 35.8 | 21.0 | -14.8 |  |
|  | *21.7 (31/143)* | *11.2 (16/143)* | *-10.5* | *17.5 (21/120)* | *1.6 (2/124)* | *-15.9* | -5.4 |
| People drinking alcohol in public places | 63.0 | 58.9 | -4.1 | 50.8 | 39.0 | -11.8 |  |
|  | *42.5 (62/146)* | *19.2 (28/146)* | *-23.3* | *24.6 (31/126)* | *10.6 (13/123)* | *-14.0* | 9.3 |
| Burglaries | 39.3 | 37.5 | -1.8 | 29.1 | 16.4 | -12.7 |  |
|  | *9.0 (13/145)* | *4.2 (6/144)* | *4.8* | *11.1 (13/117)* | *0.8 (1/122)* | *-10.3* | -15.1 |
| People hanging around | 59.5 | 60.3 | 0.8 | 52.7 | 48.0 | -4.7 |  |
|  | *39.2 (58/148)* | *24.0 (35/146)* | *-15.2* | *21.7 (28/129)* | *12.8 (16/125)* | *-8.9* | 6.3 |
| The people round here | 36.7 | 35.4 | -1.3 | 26.4 | 25.6 | -0.8 |  |
|  | *10.2 (15/147)* | *5.6 (8/144)* | *-4.6* | *10.1 (13/129)* | *2.4 (3/125)* | *-7.7* | -3.1 |
| Domestic abuse | 22.7 | 23.5 | 0.8 | 16.7 | 15.0 | -1.7 |  |
|  | *5.5 (6/110)* | *4.4 (6/136)* | *-1.1* | *2.8 (3/108)* | *2.5 (3/120)* | *-0.3* | 0.8 |
| Disturbance by children/youngsters | 58.1 | 63.7 | 5.6 | 50.4 | 59.2 | 8.8 |  |
|  | *23.0 (34/148)* | *15.8 (23/146)* | *-7.2* | *20.2 (26/129)* | *16.0 (20/125)* | *-4.2* | 3.0 |
| Nuisance from dogs | 44.2 | 52.7 | 8.5 | 38.0 | 46.0 | 8.0 |  |
|  | *21.1 (31/147)* | *19.9 (29/146)* | *-1.2* | *16.3 (21/129)* | *15.1 (19/126)* | *-1.2* | 0.0 |
