# Supplementary material for: The SHARP study: a quantitative and qualitative evaluation of the short-term outcomes of housing and neighbourhood renewal
Source: BMC Public Health. 2009 Nov 17;9:415. doi: 10.1186/1471-2458-9-415 (PMC2784462; doi:10.1186/1471-2458-9-415)
Supplement: Supplementary file 2 [file 1471-2458-9-415-S1.DOC]

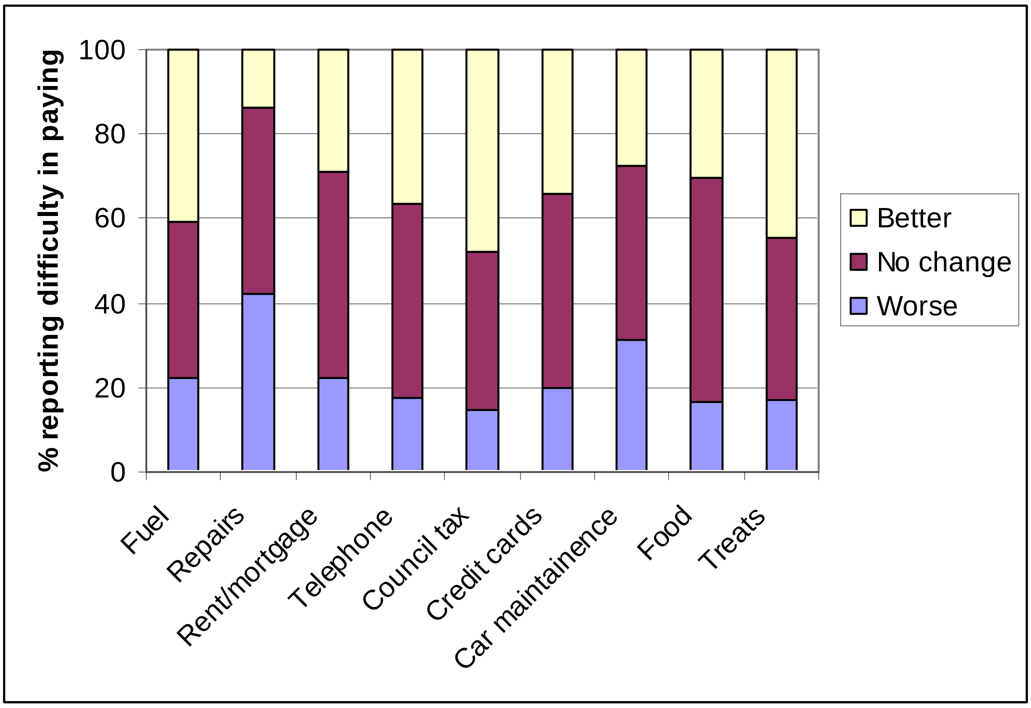
% reporting difficulty in paying
100
80
60
40
20
0
Fuel
Repairs
Rent/mortgage
Telephone
Council tax
Credit cards
Car maintainence
Food
Treats
Better
No change
Worse
